# Efficacy of Omega-3 Intake in Managing Dry Eye Disease: A Systematic Review and Meta-Analysis of Randomized Controlled Trials

**DOI:** 10.3390/jcm12227026

**Published:** 2023-11-10

**Authors:** Wei-Xiang Wang, Mei-Lan Ko

**Affiliations:** 1 School of Medicine, College of Medicine, Taipei Medical University, Taipei 110, Taiwan; b101106012@tmu.edu.tw; 2 Department of Ophthalmology, National Taiwan University Hospital, Hsin-Chu Branch, Hsin-Chu City 300, Taiwan; 3 Department of Ophthalmology, College of Medicine, National Taiwan University, Taipei 110, Taiwan

**Keywords:** dry eye disease, fatty acid, omega-3, eicosapentaenoic acid percentage, systematic review, meta-analysis

## Abstract

To explore the efficacy of omega-3 fatty acids (FAs) on patients suffering from dry eye disease (DED), a complex inflammatory condition, we reviewed data from PubMed, Embase, ClinicalTrials.gov, Web of Science, and Cochrane CENTRAL in the past 10 years (2013 to 2023). These sources provided randomized clinical trials (RCTs) that examined the efficacy of omega-3 FAs on DED patients with accessible pre- and post-intervention data, excluding trials with overlapping participants, without omega-3 supplementation, or those lacking placebo control or quantitative assessments. Two independent reviewers extracted data related to dry eye symptom scores, tear break-up time (TBUT), Schirmer’s tests, osmolarity, and corneal fluorescein staining (CFS), and the results were analyzed by Comprehensive Meta-Analysis software version 4. We incorporated 19 related RCTs assessed by the Cochrane Risk of Bias tool, encompassing 4246 DED patients with various etiologies. Patients given omega-3 treatment demonstrated more significant improvements in dry eye symptoms (Hedges’ g = −1.047; *p* < 0.001), TBUT [standardized mean difference (SMD) = −0.939; *p* < 0.001], scores from the Schirmer test (SMD = −0.372; *p* < 0.001), CFS (SMD = −0.299; *p* = 0.037), and osmolarity (SMD = −0.721; *p* < 0.001) compared to those on a placebo regimen. In the meta-regression analysis of DED symptoms, the daily dose of omega-3 (coefficient = −0.0005, *p* = 0.002), duration of omega-3 intake (coefficient = −0.1399, *p* = 0.021), and percentage of eicosapentaenoic acid (EPA) (coefficient = −0.0154, *p* < 0.001) exhibited a significant positive correlation with a reduction in dry eye symptom scores. Apart from CFS, similar trends were noted in TBUT, Schirmer tests, and osmolarity scores. Based on the evidence, omega-3 FAs effectively reduce DED symptoms, especially in high doses, for a long duration, and with increased EPA levels. However, given the heterogeneity in study results and diverse patient characteristics, caution is needed in generalizing these findings. In conclusion, omega-3 FA supplementation is still recommended for DED management in clinical settings.

## 1. Introduction

Dry eye disease (DED) is a widespread ocular disorder with multiple contributing factors characterized by inadequate tear production, suboptimal tear quality, or accelerated tear evaporation [1,2]. The pathogenesis of DED involves tear film imbalances and ocular surface inflammation, which can lead to clinical symptoms such as ocular dryness, excessive tearing, photophobia, and blurred vision [3]. The current management of DED emphasizes improving tear quality, addressing inflammation, and instituting dietary and lifestyle changes. As a first-line treatment, tear substitutes provide temporary relief; however, they cannot resolve the root causes of inflammation. Although topical corticosteroids can target this condition, their long-term side effects, including cataracts and glaucoma, restrict their use. Topical cyclosporine A is another option; nevertheless, its variable effectiveness and restricted availability present challenges [4]. Based on clinical evidence, dietary essential fatty acids (FAs) have demonstrated potential efficacy, especially in addressing inflammatory reactions. Research has indicated that omega-3 FAs may alter the composition of the lacrimal gland and enhance lacrimal secretion [5,6].

Omega-3 FAs are crucial polyunsaturated FAs that humans must acquire from food sources because they cannot produce them internally. These FAs are classified as short-chain (e.g., alpha-linolenic acid) and long-chain (e.g., docosahexaenoic acid [DHA] and eicosapentaenoic acid [EPA]). Soybeans, walnuts, chia seeds, and flaxseeds are prominent sources of short-chain omega-3 fatty acids in the plant kingdom. In the realm of seafood, fatty fish like salmon and mackerel are teeming with long-chain omega-3 fatty acids. These essential fats can also be endogenously produced by the human body through a biochemical process that involves the elongation and desaturation of short-chain fatty acids [7]. By serving as a competitive enzyme to arachidonic acids like 5-lipoxygenase and cyclooxygenases, omega-3 FAs can shift the balance of eicosanoid production toward a less inflammatory state [8].

A variety of randomized clinical trials (RCTs) have examined dry eye symptom scores, tear film break-up time (TBUT), scores of the Schirmer test, corneal fluorescein staining (CFS), and osmolarity to evaluate the efficacy of omega-3 on DED. However, variations in clinical outcomes have been observed across studies. Among these, the well-known DRy Eye Evaluation And Management (DREAM) study considered the potential influence of elements, such as patient selection, treatment limitations, and treatment duration, on the variability and differences in outcomes [9]. Given these controversies, we included additional articles published after 2018 to reflect recent developments in fish oil products and offered a more comprehensive measure for dry eye syndrome. At the same time, we did not restrict the etiology of DED to align with real-world scenarios. The meta-regression analyses were individually performed on treatment duration, omega-3 dosage, and EPA percentage to evaluate the efficacy. This systematic review and meta-analysis assessed the efficacy of omega-3 FAs in managing DED.

## 2. Materials and Methods

### 2.1. General Guidelines

The meta-analysis was conducted following the 2020 PRISMA guidelines, which set the standard for reporting systematic reviews and meta-analyses (Supplementary Table S1) [10]. The study was recorded on the International Platform of Registered Systematic Review and Meta-Analysis Protocols (i.e., “INPLASY”) under the registration number INPLASY 202390083 and did not require approval from an ethics review board or informed written consent from participants.

### 2.2. Inclusion and Exclusion Criteria

Inclusion criteria for this study were as follows: human-involved RCTs; RCTs that provided quantitative assessments before and after omega-3 intake; studies published in the past 10 years (2013 to 2023); trials that used a placebo control (without any age or treatment duration restrictions); and studies offering accessible information on dry eye evaluations both before and after the intervention or any alterations in the outcome measures.

Exclusion criteria for this study were as follows: participants were not randomized; overlapping participants; not focused on omega-3 supplementation; combination with other treatments; and not placebo-controlled.

In the present meta-analysis, population, intervention, comparison, and outcome (i.e., “PICO”) were defined as: P, human patients with DED; I, omega-3 intake; C, placebo or non-omega-3 oil; and O, alterations in the scores of dry eye symptoms, TBUT, Schirmer test, osmolarity, and CFS.

### 2.3. Search Strategy and Study Selection

The literature search for this study was independently performed by two authors using PubMed, Embase, ClinicalTrials.gov, Web of Science, and Cochrane CENTRAL electronic databases in the past 10 years (until 16 March 2023). Keywords included (“keratoconjunctivitis sicca” OR “dry eye” OR “dry eye disease”) AND (“omega-3” OR “fatty acid” OR “n − 3”). Additionally, the reference lists of any retrieved review articles were examined manually to ensure a comprehensive search for relevant studies based on the eligible criteria [11,12]. The process of screening and reviewing involved eliminating duplicates, screening titles and abstracts, and conducting a full-text review.

### 2.4. Evaluation of Methodological Quality

The Cochrane Risk of Bias tool (version 2, RoB 2, London, UK) was used to assess the methodological quality of the included studies. This tool comprises six primary elements for assessing the quality of the study: randomization process, intervention adherence, missing outcome data, outcome measurement, selective reporting, and overall risk of bias [13].

### 2.5. Data Extraction

Data from the chosen studies were extracted by two authors, including demographic information, study design, specifics of the omega-3 and placebo treatments, and outcome values. If multiple time points for post-treatment data were available, the results at the intervention’s conclusion were used for statistical evaluation. Data extraction and conversion, along with consolidation of results from distinct study arms using varying omega-3 dosages, were performed following the Cochrane Handbook for Systematic Reviews of Interventions guidelines and related medical publications [14,15]. The results for evaluating DED outcomes encompassed four subjective measures: TBUT, Schirmer test scores, CFS, and osmolarity, and two objective indicators: Dry Eye Severity Score and OSDI scores. These continuous data were gathered as mean values along with their standard deviations (SDs). If RCTs provided standard errors (SEs), SDs were calculated using the formula (SE = SD/√N) and the sample size.

### 2.6. Statistical Analysis

Owing to the diverse target groups in the studies reviewed, the present meta-analysis was performed using a random-effects model [16], facilitated by Comprehensive Meta-Analysis software version 4 (Biostat, Englewood, NJ, USA). Differences with a two-tailed *p* < 0.05 were considered to be statistically significant.

Study outcomes, such as changes in dry eye symptoms and CFS scores, were quantified using the Hedges’ g statistic along with the associated 95% confidence intervals (CIs). Effect sizes with Hedges’ g values of 0.2, 0.5, and 0.8 were considered as small, moderate, and large, respectively [17]. Other outcomes, such as TBUT, Schirmer’s test, and osmolarity, were assessed using standardized mean difference (SMD) along with the associated 95% CI. Effect sizes with SMD values of 0.2, 0.5, and 0.8 were considered as small, moderate, and large, respectively.

To determine the level of heterogeneity between the studies, I2 and Cochran’s Q statistics were utilized. Heterogeneity was classified as low, moderate, or high at I2 values of 25%, 50%, and 75%, respectively [18]. Meta-regression analysis focused on the efficacy of daily omega-3 dosage, EPA percentage of omega-3, and different treatment durations to determine whether the symptom-reducing effects of omega-3 were associated with these factors.

In the forest plot of meta-analysis, the different sizes of symbols represent the “relative weight” each holds in the respective analysis, and the “pooled” row provides a statistical summary of all study results to assess the overall effect and its credibility. All forest plots were generated by the random effect model. To ensure the reliability of the meta-analysis, sensitivity analyses were conducted with the “leave-one-out” method. This method assessed whether excluding a specific trial from the analysis led to a statistically significant change in the overall effect size [19]. Potential publication bias was assessed according to the guidelines established in the Cochrane Handbook for Systematic Reviews of Interventions [20]. Funnel plots were created and examined visually.

## 3. Results

### 3.1. Study Selection and Characteristics

**Figure 1:**
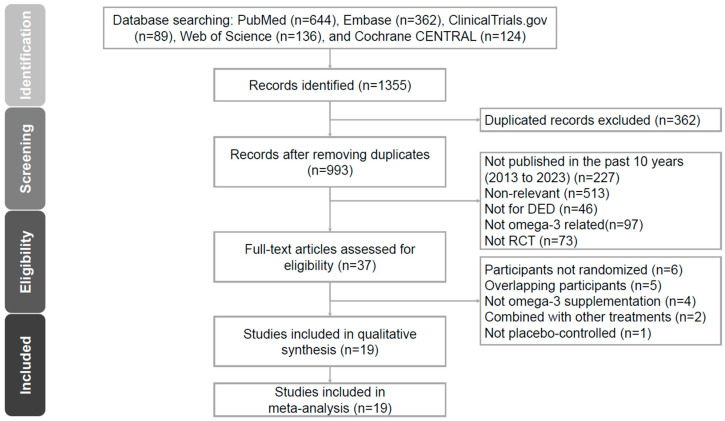
PRISMA 2020 flowchart for the current meta-analysis.

A PRISMA flow diagram illustrating the literature search process is presented in Figure 1. Several studies were excluded for the following reasons: participants were not randomized; overlapping participants; not focused on omega-3 supplementation; combination with other treatments; and not placebo-controlled. Ultimately, 19 RCTs met the inclusion criteria and were chosen for the analysis.

The 19 included RCTs comprised a total of 4246 subjects (mean [± standard deviation] age, 48.4 ± 13.1 years; 42.6% male) [2,5,6,9,21,22,23,24,25,26,27,28,29,30,31,32,33,34,35]. Study durations ranged from 1 to 12 months [6,9,32]. The etiology of dry eye among the subjects included meibomian gland dysfunction [23,25,28], contact lens-associated [5], visual display terminal syndrome [24,27,35], rosacea [26], laser-assisted in situ keratomileusis (LASIK)-associated [31] and cataract surgery-associated [33] factors, and not specified [2,6,9,21,22,29,32,34]. Specifics of the interventions in the selected trials, including EPA percentage, follow-up duration, and omega-3 dosage, are summarized in Table 1.

### 3.2. Quality Assessment of the Selected Studies

**Figure 2:**
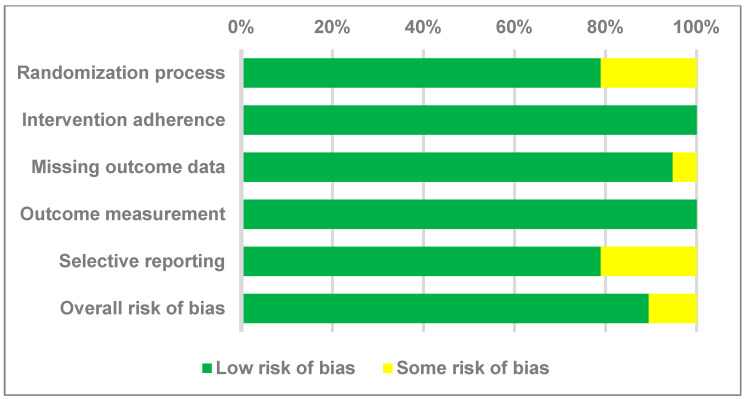
Summary of quality assessment of studies included in the meta-analysis using Cochrane Risk of Bias 2 tool.

Regarding the overall risk of bias, the analysis revealed that 84.2% of the studies demonstrated a low bias risk, 15.8% demonstrated some bias risk, and none (0%) demonstrated a high bias risk (Figure 2). On thorough evaluation, four studies were classified as exhibiting “some” risk of bias in their randomization procedures due to a lack of disclosure regarding allocation concealment [21,23,25,29]. One study was classified as having “some” risk of bias in missing outcome data because the study did not provide sufficient outcome data to support statistical consequences [23]. Four studies were classified as having “some” risk of bias in selective reporting due to a lack of clarity regarding selective reporting [22,25,26,30]. The summary of the risk of bias analysis can be found in Supplementary Table S2.

### 3.3. Efficacy of Omega-3 Supplementation on Different Outcomes

**Figure 3:**
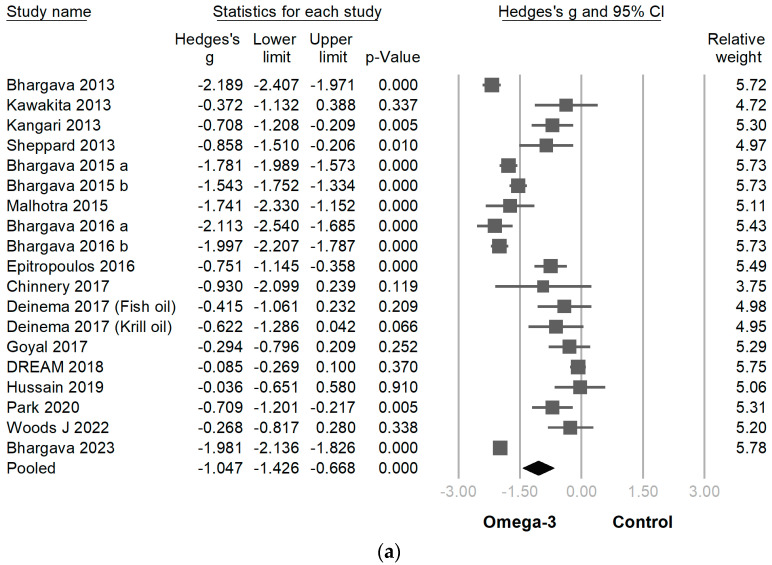

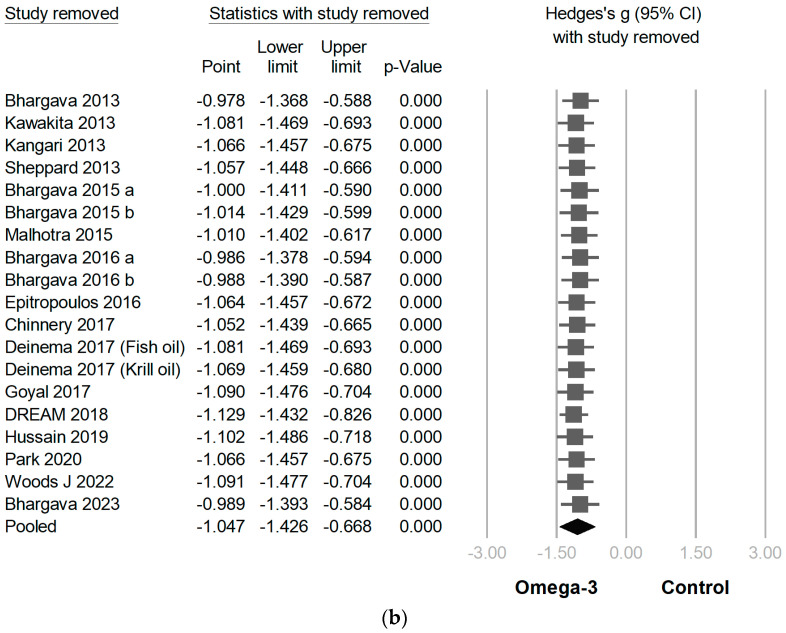
(**a**) Forest plot analysis comparing omega-3 effects on dry eye symptoms to placebo: Omega-3 showed efficacy in diminishing dry eye symptoms. CI, confidence interval. (**b**) A sensitivity examination was conducted using the method of one-study removal. This examination revealed that the efficacy of omega-3 on reducing dry eye symptoms remained statistically significant consistently across the analysis [2,5,6,9,21,22,24,25,26,27,28,29,30,31,32,33,34,35].

All comparative data for dry eye syndrome outcomes were obtained at the end of the period specified in Table 1. In 12 studies, the Ocular Surface Disease Index questionnaire was performed to evaluate dry eye symptoms, while the Dry Eye Severity Score questionnaire was employed for the same purpose in 6 other studies. In 18 of the 19 RCTs, omega-3 supplementation led to a statistically significant decline in DED symptoms (Hedges’ g −1.047 [95% CI −0.668~−1.426]; *p* < 0.001; I² = 96.1%) (Figure 3a). Nevertheless, the notable presence of high heterogeneity was observed. Therefore, a sensitivity examination was conducted with the leave-one-out method. The results revealed that the efficacy of omega-3 supplementation on the reduction of dry eye symptoms remained consistently statistically significant across the analysis. Importantly, the overall effect sizes maintained their statistical significance regardless of the exclusion of any specific study (Figure 3b).

**Figure 4:**
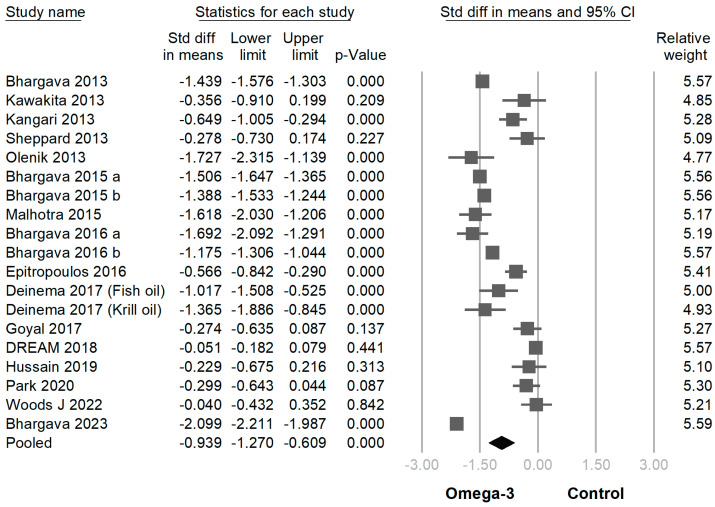
Forest plot analysis comparing omega-3 effects on tear break-up time to placebo: Omega-3 showed efficacy in prolonging tear break-up time [2,5,6,9,21,22,23,24,25,26,27,28,30,31,32,33,34,35].

**Figure 5:**
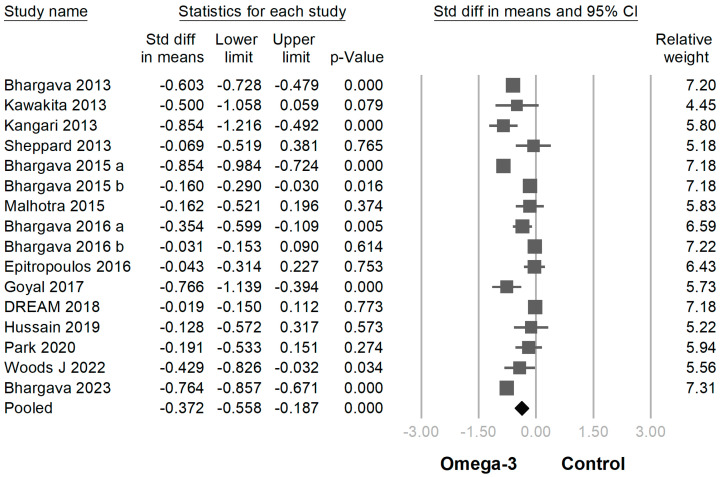
Forest plot analysis comparing omega-3 effects on Schirmer test to placebo: Omega-3 showed greater improvement in Schirmer test [2,5,6,9,21,22,24,25,26,27,28,31,32,33,34,35].

**Figure 6:**
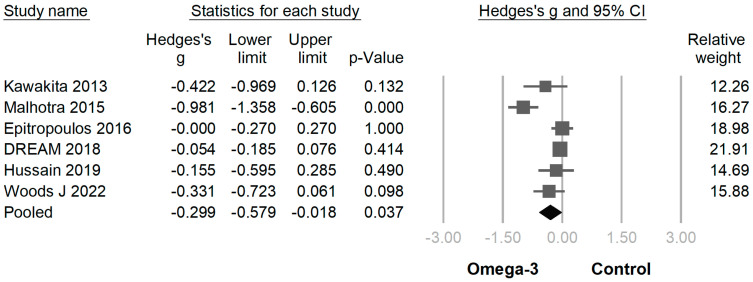
Forest plot analysis comparing omega-3 effects on corneal fluorescein staining to placebo: Omega-3 showed displayed a statistically significant decline in corneal fluorescein staining [9,21,25,28,32,34].

**Figure 7:**
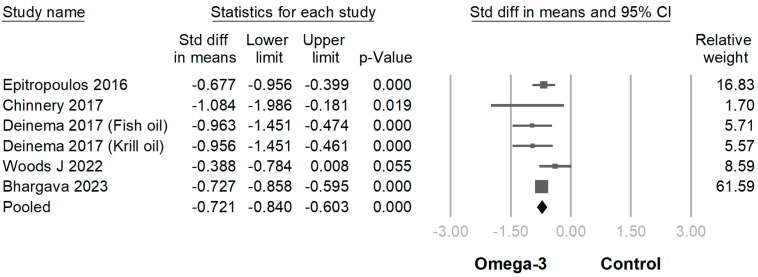
Forest plot analysis comparing omega-3 effects on osmolarity of the ocular surfaces to placebo: Omega-3 showed exhibited statistically significant decrease in osmolarity [28,29,30,34,35].

In 18 of 19 trials, the omega-3 supplementation group exhibited statistically higher TBUT compared with the placebo group (SMD −0.939 [95% CI −0.609~−1.270]; *p* < 0.001) (Figure 4). In 16 of the 19 RCTs, the omega-3 supplementation group exhibited a statistically greater improvement in the Schirmer test than the placebo group (SMD −0.372 [95% CI −0.187~−0.558]; *p* < 0.001) (Figure 5). In 6 of the 19 trials, the omega-3 supplementation group exhibited a statistically significant decline in CFS (SMD −0.299 [95% CI −0.018~−0.579]; *p* = 0.037) (Figure 6). In 5 of the 19 trials, the omega-3 supplementation group exhibited a statistically significant decrease in the osmolarity of the ocular surfaces compared with the placebo group (SMD −0.721 [95% CI −0.603~−0.840]; *p* < 0.001) (Figure 7).

### 3.4. Publication Bias and Meta-Regression

Funnel plot analysis was performed for dry eye symptoms (*p* = 0.101), TBUT (*p* = 0.169), Schirmer’s test (*p* = 0.513), CFS (*p* = 0.205), and osmolarity (*p* = 0.629). Based on the Egger’s test *p*-value, results indicated no evidence of publication bias for these five prognostic indicators (Table 2). Meanwhile, meta-regression was used to investigate whether factors, such as treatment duration, daily dosage of omega-3 fatty acids, and percentage of EPA, influenced the outcomes of dry eye symptoms TBUT, Schirmer test, CFS, and osmolarity (Table 2). Meta-regression was considered to be significant at *p* < 0.05. First, a meta-regression for treatment duration was performed using months as the unit. The results of the analysis revealed significant differences in the scores for dry eye symptoms (coefficient = −0.1399, *p* = 0.021), TBUT (coefficient = −0.1297, *p* = 0.009), Schirmer’s test (coefficient = −0.0476, *p* = 0.018), and osmolarity (coefficient = −0.1799, *p* < 0.001). This implies that the longer the duration of omega-3 intake, the better the observed efficacy in these categories, except for CFS (coefficient = −0.0224, *p* = 0.337).

Second, meta-regression for omega-3 daily doses was performed using milligrams as the unit. The results revealed significant differences in the scores for dry eye symptoms (coefficient = −0.0005, *p* = 0.002), TBUT (coefficient = −0.0004, *p* = 0.001), Schirmer’s test (coefficient = −0.0002, *p* = 0.015), and osmolarity (coefficient = −0.0004, *p* < 0.001). These findings imply that with an increased dosage of omega-3, there was a corresponding enhancement in outcome(s), except for CFS (coefficient = −0.0001, *p* = 0.205). Finally, meta-regression for EPA percentage was performed. Significant differences were found in the scores for dry eye symptoms (coefficient = −0.0154, *p* < 0.001), TBUT (coefficient = −0.0138, *p* < 0.001), Schirmer’s test (coefficient = −0.0058, *p* < 0.001), and osmolarity (coefficient = −0.0104, *p* < 0.001). These results suggest that higher EPA intake leads to improved outcomes in these parameters. However, no such correlation was observed for CFS (coefficient = −0.004, *p* = 0.05).

## 4. Discussion

The present meta-analysis included 19 RCTs from the most recent decade, enrolling 4246 individuals with different types of DED, and demonstrated that omega-3 significantly alleviated dry eye symptoms, as confirmed by dry eye symptom scores, TBUT, Schirmer test, osmolarity, and CFS. Statistical significance remained consistent across the sensitivity analyses, and the data reported in Table 2 confirm the absence of publication bias (Supplementary Figure S1). In the meta-regression, treatment duration, daily dosage of omega-3 FAs, and percentage of EPA were correlated with a more substantial decrease in dry eye symptoms and improvement in outcome measures. However, the lack of significance for CFS may be attributed to the various assessment standards used in the six relevant studies. These standards included the Oxford scale (three studies), National Eye Institute scale (one study), Efron scale (one study), and van Bijsterveld scale (one study).

In laboratory settings, omega-3 FAs can be converted into powerful anti-inflammatory substances known as resolvins and protectins [36]. These substances help reduce inflammation by lowering the production of numerous proinflammatory cytokines in various parts of the body, including the eyes [36]. In an in vivo study, Bhargava et al. found that oral supplementation with omega-3 FAs improved tear stability, which improved the quality of the oily layer and reduced tear evaporation [24]. Oleñik et al. reported that omega-3 FAs appeared to affect DED by rejuvenating the lipid layer in the tear film through the resolution of meibomian gland dysfunction and enhancing tear production from the lacrimal gland [23]. Epitropoulos et al. suggested that the efficacy of omega-3 FAs arises from both their anti-inflammatory properties and their effect on the lipid composition generated by the epithelial cells of the meibomian gland. Similar conclusions were reported in other RCTs [23,25,28].

To our knowledge, the current study was the most comprehensive meta-analysis examining the efficacy of omega-3 FA intake in the management of DED. To reflect real-world conditions, we analyzed DED data for various etiologies, including visual display terminal syndrome, rosacea, and meibomian gland dysfunction, as well as the impact of contact lens wear, LASIK, and cataract surgeries [5,31,33]. In 2014, Liu et al. found that supplementation with omega-3 FAs enhanced Schirmer test and TBUT scores, although it did not significantly alleviate symptoms of ocular discomfort compared with placebo [37]. In 2019, two separate meta-analyses by Giuseppe et al. and Sheng-Chu et al. suggested that supplementation with omega-3 FAs could enhance dry eye symptom relief, stabilize the tear film, and augment tear production in DED patients [11,12]. Given the current evidence, it is important to note that the therapeutic efficacy of omega-3 FAs may differ across various regions of the world, potentially due to the influence of regional dietary patterns [11].

Despite these insights, these studies were unable to establish a clear correlation between the efficacy of the treatment and the dosage or duration of supplementation. These points, including the daily dosage of omega-3 FA, duration of intake, and proportion of EPA, were highlighted in the meta-regression analysis. In the meta-regression analysis of DED symptoms, the daily dose of omega-3, duration of omega-3 intake, and percentage of EPA exhibited a significant positive correlation with a reduction in dry eye symptom scores (Supplementary Figure S2). Apart from CFS, similar trends were noted in TBUT, Schirmer tests, and osmolarity scores. These findings confirm that with an increased dosage (maximum, 3000 mg), longer duration of intake (maximum, 12 months), and a higher proportion of EPA (maximum, 80% in the study), there was a notable alleviation of DED. Nevertheless, discerning the optimal values from this analysis is challenging.

However, this meta-analysis had several limitations that must be acknowledged. First, significant heterogeneity was evident in all outcome measures, suggesting inconsistencies among the study results. Although we used both meta-regression and sensitivity analyses, unexplained variances remained. Second, there were notable differences in patient characteristics across the included trials, including age, sex, criteria for diagnosing dry eye, and its underlying causes, all of which potentially affected the analysis of the efficacy of omega-3 supplementation. Third, although we identified correlations between efficacy and duration, dosage, and EPA percentage, the included studies only tracked outcomes for up to 12 months, with dosages reaching a maximum of 3000 mg and EPA percentages capped at 80%. Therefore, the efficacy of omega-3 above these limits remains uncertain. Finally, the dietary intake of omega-3 metabolites by individuals in the study is unknown, and there are other metabolites, such as alpha-linolenic acid (ALA), whose effects are not clearly understood in the current application. Despite these limitations, the current meta-analysis still reveals impressive benefits of omega-3 supplementation, likely reflecting the real-world scenario of participants with a diverse diet and omega-3 supplementation.

## 5. Conclusions

The findings from this meta-analysis underscore the efficacy of omega-3 supplementation in managing DED. Omega-3 FAs consistently alleviated DED symptoms, particularly at high doses, with prolonged intake, and with increased EPA levels. Given the present evidence, omega-3 FA supplementation is suggested for clinical use in the management of DED. Although these results reinforce the therapeutic potential of omega-3 FAs, the analysis revealed some heterogeneity across the studies and emphasized the variations in patient characteristics. These limitations highlight the need for future research. Longer-term studies and broader dosage ranges may pave the way for refined clinical guidelines and enhanced therapeutic strategies for the treatment of DED.

## Figures and Tables

**Table 1:** Intervention details of the included trials.

Author (Year of Publication)	Etiology of Dry Eye Disease	Sample Size		Age		Sex (M/F)		Duration (Month)	Omega-3 Fatty Acid Daily Dose	EPA Percentage (%)
		Omega-3	Placebo	Omega-3	Placebo	Omega-3	Placebo			
Bhargava 2013 [2]	Not specified	264	254	38.8	40.1	Total: 254/268		3	EPA 650 mg + DHA 350 mg	65
Kawakita 2013 [21]	Not specified	15	11	52.5	51.9	5/10	1/10	4	EPA 1245 mg + DHA 540 mg	70
Kangari 2013 [6]	Not specified	33	31	60.6	61.8	15/18	11/20	1	EPA 360 mg + DHA 240 mg	60
Sheppard 2013 [22]	Not specified	19	19	62	61	0/19	0/19	6	EPA 128 mg + DHA 99 mg + other fatty acid 1185 mg	9
Oleñik 2013 [23]	MGD	30	31	58	54	9/24	9/22	3	EPA 127.5 mg + DHA 1050 mg	11
Bhargava 2015 a [5]	Contact lens	240	256	Not specified		Not specified		6	EPA 720 mg + DHA 480 mg	60
Bhargava 2015 b [24]	Visual display terminal users	220	236	22.8	23.7	Total: 219/237		3	EPA 360 mg + DHA 240 mg	60
Malhotra 2015 [25]	MGD	30	30	53.3	53.6	13/17	19/11	3	EPA 720 mg + DHA 480 mg	60
Bhargava 2016 a [26]	Rosacea	65	65	47.7	48.9	25/40	27/38	6	EPA 720 mg + DHA 480 mg	60
Bhargava 2016 b [27]	Visual display terminal users	256	266	28.9	29.6	Not specified		1.5	EPA 1440 mg + DHA 960 mg	60
Epitropoulos 2016 [28]	MGD	54	51	57	56.5	16/38	14/37	3	EPA 1680 mg + DHA 560 mg	75
Chinnery 2017 [29]	Not specified	8	4	42	46	2/6	1/3	3	EPA 1000 mg + DHA 500 mg	67
Deinema 2017 [30]	Not specified	37	17	40.8	46.2	15/22	3/14	3	Fish oil (EPA 1000 mg + DHA 500 mg)	67
									Krill oil (EPA 945 mg + DHA 510 mg)	65
Goyal 2017 [31]	LASIK-associated	30	30	23.6	23.6	Total: 27/33		3	EPA 720 mg + DHA 480 mg	60
DREAM 2018 [9]	Not specified	349	186	58.3	57.5	65/284	36/150	12	EPA 2000 mg + DHA 1000 mg	67
Hussain 2019 [32]	Not specified	22	21	58.2	58.4	3/19	4/17	12	EPA 2000 mg + DHA 1000 mg	67
Park 2020 [33]	Cataract surgery-associated	32	34	64.6	66.3	12/20	12/22	2	EPA 1680 mg + DHA 560 mg	75
Woods J 2022 [34]	Not specified	24	26	32	35	3/21	11/15	3	EPA 1200 mg + DHA 300 mg	80
Bhargava 2023 [35]	Visual display terminal users	470	480	6.5	25.8	225/255	242/238	6	EPA 1440 mg + DHA 960 mg	60

Abbreviation: M = Male; F = Female; EPA = Eicosapentaenoic Acid; DHA = Docosahexaenoic Acid; MGD = Meibomian Gland Dysfunction; LASIK = Laser-Assisted In Situ Keratomileusis.

**Table 2:** Outcomes of publication bias and meta-regression.

Outcome	Publication Bias	Meta-Regression by Duration (Month)		Meta-Regression by Omega-3 Daily Dose (mg)		Meta-Regression by EPA Percentage	
	Egger’s Test *p*-Value	Coefficient	*p*-Value	Coefficient	*p*-Value	Coefficient	*p*-Value
Score of Dry Eye Symptoms	0.101	−0.1399	0.021	−0.0005	0.002	−0.0154	<0.001
TBUT	0.169	−0.1297	0.009	−0.0004	0.001	−0.0138	<0.001
Schirmer Test	0.513	−0.0476	0.018	−0.0002	0.015	−0.0058	<0.001
CFS	0.205	−0.0224	0.337	−0.0001	0.205	−0.004	0.05
Osmolarity	0.629	−0.1799	<0.001	−0.0004	<0.001	−0.0104	<0.001

Abbreviation: TBUT = Tear Break-Up Time; CFS = Corneal Fluorescein Staining; EPA = Eicosapentaenoic Acid.

## Data Availability

Data are contained within the article.

## Supplementary Materials

The following supporting information can be downloaded at: https://www.mdpi.com/article/10.3390/jcm12227026/s1, Table S1: PRISMA 2020 Checklist, Table S2: Detailed quality assessment of included studies using Cochrane Risk of Bias 2 tool, Figure S1: Funnel plot on the scores of dry eye symptom. Figure S2: Meta-regression analysis for dry eye symptom score on omega-3 daily dose, duration of omega-3 intake, and EPA percentage.
