# Supplementary material for: Efficacy of Omega-3 Intake in Managing Dry Eye Disease: A Systematic Review and Meta-Analysis of Randomized Controlled Trials
Source: J Clin Med. 2023 Nov 10;12(22):7026. doi: 10.3390/jcm12227026 (PMC10672334; doi:10.3390/jcm12227026)
Supplement: Supplementary file 1 [file jcm-12-07026-s001.zip › jcm-2661411-supplementary.pdf]

Table S1. PRISMA 2020 Checklist

Section and Topic	Item #	Checklist item	Location where item is reported
**TITLE**			
Title	1	Identify the report as a systematic review.	Title (Page 1)
**ABSTRACT**			
Abstract	2	See the PRISMA 2020 for Abstracts checklist.	Abstract (Page 1)
**INTRODUCTION**			
Rationale	3	Describe the rationale for the review in the context of existing knowledge.	Introduction (Page 1-2)
Objectives	4	Provide an explicit statement of the objective(s) or question(s) the review addresses.	Introduction (Page 1-2)
**METHODS**			
Eligibility criteria	5	Specify the inclusion and exclusion criteria for the review and how studies were grouped for the syntheses.	Methods (Line 86-99)
Information sources	6	Specify all databases, registers, websites, organisations, reference lists and other sources searched or consulted to identify studies. Specify the date when each source was last searched or consulted.	Methods (Line 100-105)
Search strategy	7	Present the full search strategies for all databases, registers and websites, including any filters and limits used.	Methods (Line 100-105)
Selection process	8	Specify the methods used to decide whether a study met the inclusion criteria of the review, including how many reviewers screened each record and each report retrieved, whether they worked independently, and if applicable, details of automation tools used in the process.	Methods (Line 105-108)
Data collection process	9	Specify the methods used to collect data from reports, including how many reviewers collected data from each report, whether they worked independently, any processes for obtaining or confirming data from study investigators, and if applicable, details of automation tools used in the process.	Methods (Line 105-108)
Data items	10a	List and define all outcomes for which data were sought. Specify whether all results that were compatible with each outcome domain in each study were sought (e.g. for all measures, time points,	Methods (Line 115-127)

Section and Topic	Item #	Checklist item	Location where item is reported
		analyses), and if not, the methods used to decide which results to collect.	
	10b	List and define all other variables for which data were sought (e.g. participant and intervention characteristics, funding sources). Describe any assumptions made about any missing or unclear information.	Methods (Line 115-127)
Study risk of bias assessment	11	Specify the methods used to assess risk of bias in the included studies, including details of the tool(s) used, how many reviewers assessed each study and whether they worked independently, and if applicable, details of automation tools used in the process.	Methods (Line 109-114)
Effect measures	12	Specify for each outcome the effect measure(s) (e.g. risk ratio, mean difference) used in the synthesis or presentation of results.	Methods (Line 115-127)
Synthesis methods	13a	Describe the processes used to decide which studies were eligible for each synthesis (e.g. tabulating the study intervention characteristics and comparing against the planned groups for each synthesis (item #5)).	Methods (Line 128-151)
	13b	Describe any methods required to prepare the data for presentation or synthesis, such as handling of missing summary statistics, or data conversions.	Methods (Line 128-151)
	13c	Describe any methods used to tabulate or visually display results of individual studies and syntheses.	Methods (Line 128-151)
	13d	Describe any methods used to synthesize results and provide a rationale for the choice(s). If meta-analysis was performed, describe the model(s), method(s) to identify the presence and extent of statistical heterogeneity, and software package(s) used.	Methods (Line 128-151)
	13e	Describe any methods used to explore possible causes of heterogeneity among study results (e.g. subgroup analysis, meta-regression).	Methods (Line 128-151)
	13f	Describe any sensitivity analyses conducted to assess robustness of the synthesized results.	Methods (Line 128-151)
Reporting bias assessment	14	Describe any methods used to assess risk of bias due to missing results in a synthesis (arising from reporting biases).	Methods (Line 109-114)

Section and Topic	Item #	Checklist item	Location where item is reported
Certainty assessment	15	Describe any methods used to assess certainty (or confidence) in the body of evidence for an outcome.	Methods (Line 128-151)
**RESULTS**			
Study selection	16a	Describe the results of the search and selection process, from the number of records identified in the search to the number of studies included in the review, ideally using a flow diagram.	Results (Line 153-158), Figure 1
	16b	Cite studies that might appear to meet the inclusion criteria, but which were excluded, and explain why they were excluded.	Results (Line 153-158), Figure 1
Study characteristics	17	Cite each included study and present its characteristics.	Results (Line 161-168), Table 1
Risk of bias in studies	18	Present assessments of risk of bias for each included study.	Results (Line 172-186), Table S2, Figure 2
Results of individual studies	19	For all outcomes, present, for each study: (a) summary statistics for each group (where appropriate) and (b) an effect estimate and its precision (e.g. confidence/credible interval), ideally using structured tables or plots.	Results (Line 187-227), Figure 3-7
Results of syntheses	20a	For each synthesis, briefly summarise the characteristics and risk of bias among contributing studies.	Results (Line 187-227), Table S2
	20b	Present results of all statistical syntheses conducted. If meta-analysis was done, present for each the summary estimate and its precision (e.g. confidence/credible interval) and measures of statistical heterogeneity. If comparing groups, describe the direction of the effect.	Results (Line 187-227), Figure 3-7
	20c	Present results of all investigations of possible causes of heterogeneity among study results.	Results (Line 187-227), Figure 3-7
	20d	Present results of all sensitivity analyses conducted to assess the	Results (Line 187-227),

Section and Topic	Item #	Checklist item	Location where item is reported
		robustness of the synthesized results.	Figure 3b
Reporting biases	21	Present assessments of risk of bias due to missing results (arising from reporting biases) for each synthesis assessed.	Results (Line 228-257), Table 2
Certainty of evidence	22	Present assessments of certainty (or confidence) in the body of evidence for each outcome assessed.	Results (Line 187-227)
**DISCUSSION**			
Discussion	23a	Provide a general interpretation of the results in the context of other evidence.	Discussion (Line 258-294)
	23b	Discuss any limitations of the evidence included in the review.	Discussion (Line 295-321)
	23c	Discuss any limitations of the review processes used.	Discussion (Line 295-321)
	23d	Discuss implications of the results for practice, policy, and future research.	Discussion (Line 306-321)
**OTHER INFORMATION**			
Registration and protocol	24a	Provide registration information for the review, including register name and registration number, or state that the review was not registered.	Methods (Line 79-85)
	24b	Indicate where the review protocol can be accessed, or state that a protocol was not prepared.	Methods (Line 79-85)
	24c	Describe and explain any amendments to information provided at registration or in the protocol.	Methods (Line 79-85)
Support	25	Describe sources of financial or non-financial support for the review, and the role of the funders or sponsors in the review.	Non-financial support
Competing interests	26	Declare any competing interests of review authors.	No conflict of interest
Availability of data, code and other materials	27	Report which of the following are publicly available and where they can be found: template data collection forms; data extracted from included studies; data used for all analyses; analytic code; any other materials used in the review.	Results

Table S2. Detailed quality assessment of included studies using Cochrane risk of bias 2 tool

First Author	Randomization process	Intervention adherence	Missing outcome data	Outcome measurement	Selective reporting	Overall RoB
Bhargava 2013 [2]	L	L	L	L	L	L
Kawakita 2013 [21]	S [1]	L	L	L	L	S [3]
Kangari 2013 [6]	L	L	L	L	L	L
Sheppard 2013 [22]	L	L	L	L	S [4]	L
Oleñik 2013 [23]	S [1]	L	S [2]	L	L	S [2]
Bhargava 2015 a [5]	L	L	L	L	L	L
Bhargava 2015 b [24]	L	L	L	L	L	L
Malhotra 2015 [25]	S [1]	L	L	L	S [4]	L
Bhargava 2016 a [26]	L	L	L	L	S [4]	L
Bhargava 2016 b [27]	L	L	L	L	L	L
Epitropoulos 2016 [28]	L	L	L	L	L	L
Chinnery 2017 [29]	S [1]	L	L	L	L	S [3]
Deinema 2017 [30]	L	L	L	L	S [4]	L
Goyal 2017 [31]	L	L	L	L	L	L
DREAM 2018 [9]	L	L	L	L	L	L
Hussain 2019 [32]	L	L	L	L	L	L
Park 2020 [33]	L	L	L	L	L	L
Woods J 2022 [34]	L	L	L	L	L	L
Bhargava 2023 [35]	L	L	L	L	L	L

[1] The studies didn't provide allocation concealment details.

[2] The study didn’t provide sufficient outcome data, making it difficult to evaluate the final consequences.

[3] The insufficient sample size in these studies could lead to statistical bias.

[4] The clarity of the studies being free from selective reporting was unclear

S, some risk of bias; L, low risk of bias; RoB, risk of bias.

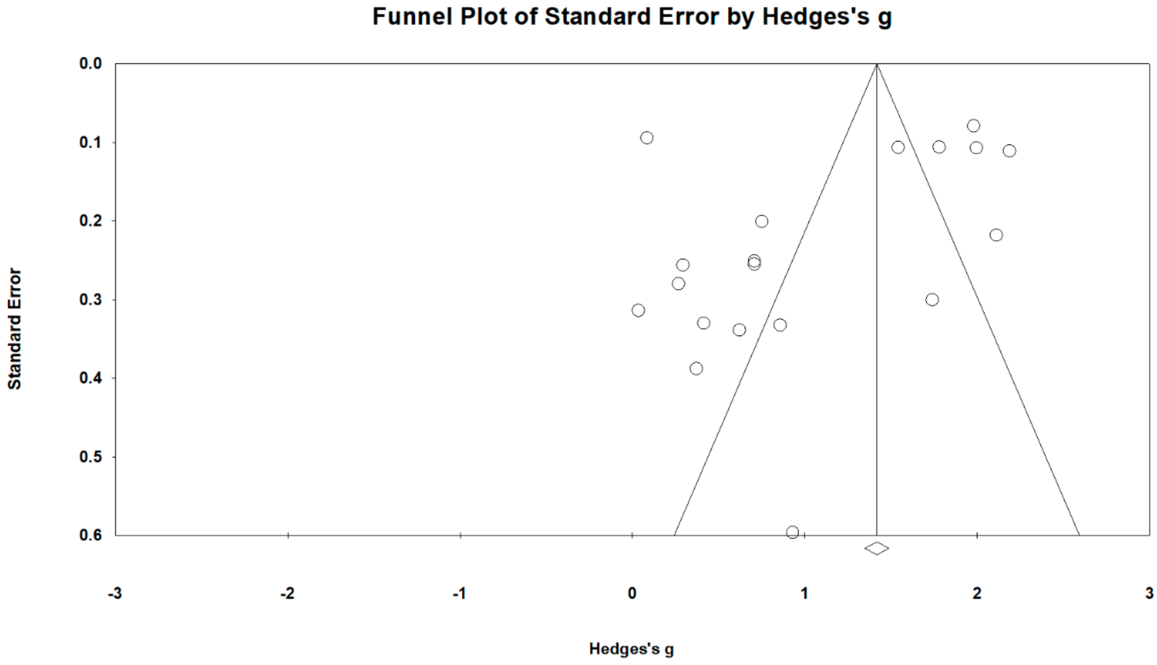

Figure S1. Funnel plot on the scores of dry eye symptom showed asymmetric distribution.

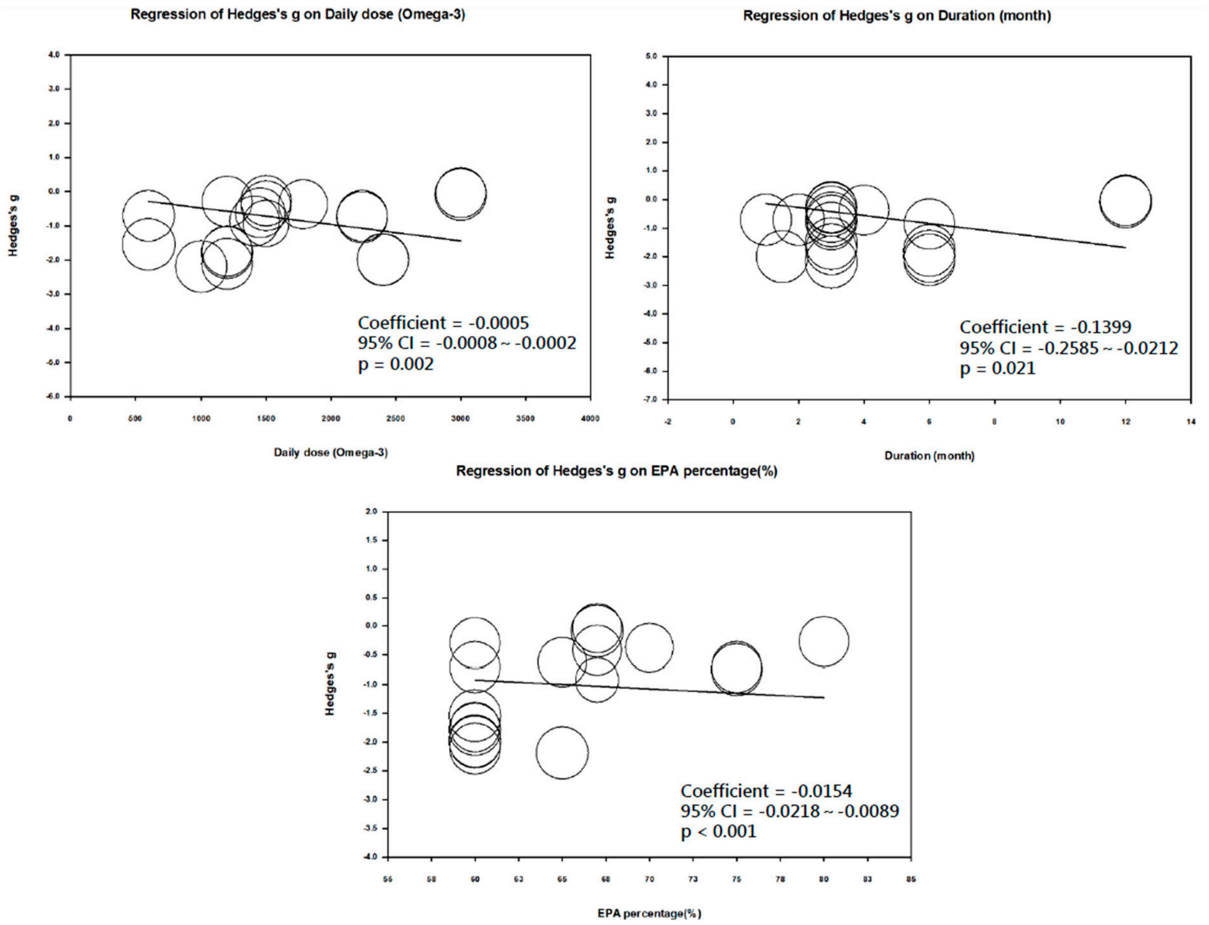

Figure S2. Meta-regression analysis for dry eye symptom score on omega-3 daily dose, duration of omega-3 intake, and EPA percentage.
